# Evaluation of lung involvement in COVID-19 pneumonia based on ultrasound images

**DOI:** 10.1186/s12938-021-00863-x

**Published:** 2021-03-20

**Authors:** Zhaoyu Hu, Zhenhua Liu, Yijie Dong, Jianjian Liu, Bin Huang, Aihua Liu, Jingjing Huang, Xujuan Pu, Xia Shi, Jinhua Yu, Yang Xiao, Hui Zhang, Jianqiao Zhou

**Affiliations:** 1 Department of Electronic Engineering, Fudan University, Shanghai, 200433 China; 2 Department of Ultrasound, Ruijin Hospital, Shanghai Jiaotong University School of Medicine, Shanghai, 200025 China; 3 Department of Ultrasound, Shanghai Public Health Clinical Center, Shanghai, 201508 China; 4 Department of Ultrasound, Xixi Hospital of Hangzhou, Hangzhou, 310023 China; 5 Department of Ultrasound, The Six Hospital of Wuhan, Affiliated Hospital of Jianghang University, Wuhan, 430015 China; 6 Institute of Biomedical and Health Engineering Shenzhen Institutes of Advanced Technology, Chinese Academy of Sciences, Shenzhen, 440305 China

**Keywords:** Ultrasound, Lung involvement, Classification, COVID-19, Neural network

## Abstract

**Background:**

Lung ultrasound (LUS) can be an important imaging tool for the diagnosis and assessment of lung involvement. Ultrasound sonograms have been confirmed to illustrate damage to a person’s lungs, which means that the correct classification and scoring of a patient’s sonogram can be used to assess lung involvement.

**Methods:**

The purpose of this study was to establish a lung involvement assessment model based on deep learning. A novel multimodal channel and receptive field attention network combined with ResNeXt (MCRFNet) was proposed to classify sonograms, and the network can automatically fuse shallow features and determine the importance of different channels and respective fields. Finally, sonogram classes were transformed into scores to evaluate lung involvement from the initial diagnosis to rehabilitation.

**Results and conclusion:**

Using multicenter and multimodal ultrasound data from 104 patients, the diagnostic model achieved 94.39% accuracy, 82.28% precision, 76.27% sensitivity, and 96.44% specificity. The lung involvement severity and the trend of COVID-19 pneumonia were evaluated quantitatively.

## Background

As of February 26, 2021, 113 509 086 confirmed cases of novel coronavirus 2019 (COVID-19) disease and 2,514,776 COVID-19-related deaths had been reported worldwide. COVID-19 has been reported in 212 countries [1]. The World Health Organization classified COVID-19 as a global pandemic [2]. Due to the increasing number of cases, it is necessary to rapidly diagnose patients and assess the severity of the disease.

The current gold standard for the diagnosis of COVID-19 is reverse transcription-polymerase chain reaction (RT-PCR) analysis of respiratory specimens. However, due to inaccurate methods when collecting samples via nasopharyngeal swabs, the false-negative rate is high [3]. Delays in the diagnosis of COVID-19 will cause the spread of the disease and the aggravation of a patient's condition. Computed tomography (CT) is the main method for diagnosing and evaluating the severity of COVID-19 pneumonia [4, 5]. However, CT also has the following problems in lung-related diagnosis. First, CT-based diagnosis is costly and involves radiation [6]. In unstable and critically ill patients, CT is not easy to perform. In addition, patients who are sensitive to radiation, such as pregnant women, need to avoid the radiation caused by CT. Second, it is of great clinical significance to determine whether there is pulmonary airway obstruction in patients with COVID-19 pneumonia. CT can only obtain static images and cannot evaluate the movement of gas in the bronchi and bronchiole in real time.

As a nonradiation medical imaging method, ultrasound is highly sensitive to the diagnosis of various lung diseases [7]. Studies have shown that lung ultrasonography (LUS) can be an important imaging tool for diagnosing common pneumonia and assessing the degree of lung involvement [8]. For example, Liu et al. proved the effectiveness of bedside LUS in the diagnosis of community-acquired common pneumonia. With CT as the gold standard, the diagnosis of community-acquired common pneumonia by LUS has reached 96.1% accuracy, and the diagnostic efficiency of LUS far exceeds that of chest X-ray [9]. LUS has been used as an effective imaging method for diagnosing common pneumonia in many institutions. The advantages of LUS are that it is inexpensive, does not involve radiation, is easy to obtain, and can be checked at the bedside, which is especially useful for patients with severe pneumonia [10, 11].

Current studies [12–17] are more focused on the diagnosis and segmentation tasks of images. Few researchers examine the impact of COVID-19 on internal organ damage among patients, especially based on ultrasound images; internal organ damage is equally important for understanding COVID-19. The current challenge for COVID-19 is not the diagnosis, but the severity and drug intake. In general, the limitations and challenges of the current research are as follows: (1) method validation of multicenter data; (2) sufficient utilization of ultrasound data; and 3) evaluation of the impact of COVID-19 on internal organs and bodily functions.

To address the issues presented above, we proposed a novel multimodal channel and receptive field attention network combined with ResNeXt (MCRFNet) for assessing lung damage in COVID-19 patients. The network can automatically fuse shallow features and determine the importance of different channels and respective fields to classify the sonograms correctly. Sonogram classes were transformed into scores to evaluate lung involvement from the initial diagnosis to rehabilitation. Proposed method can help doctors combine the scores with other indicators to evaluate the patient’s lung involvement. It is more beneficial to use this scoring system to improve the nursing level of patients with COVID-19.

## Results

### MCRFNet classification accuracy

We employed four widely used metrics to quantitatively evaluate the COVID-19 lung sonogram classification performances, including accuracy (Acc), precision (PP), sensitivity (Se), and specificity (Sp) [18, 19]. In general, a better classification performance will have higher Acc, PP, Se, and Sp. Acc describes the proportion of correctly classified images, which is expressed as follows:

1 $${\text{Acc}} = \frac{{\text{TP + TN}}}{{\text{TP + TN + FN + FP}}} \times 100\%$$

where TP, TN, FP, and FN represent the number of true-positive predictions, true-negative predictions, false-positive predictions, and false-negative predictions, respectively. PP is useful for measuring the proportion of true-positive predictions of overall positive images and is defined as:

2 $${\text{PP}} = \frac{{{\text{TP}}}}{{{\text{TP + FP}}}} \times 100\%$$

Se, which is a measure of the number of true-positive predictions and false-negative predictions, is defined as:

3 $${\text{Se}} = \frac{{{\text{TP}}}}{{\text{TP + FN}}} \times 100\%$$

Finally, Sp considers the number of true-negative predictions and false-positive predictions, which is defined as:

4 $${\text{Sp}} = \frac{{{\text{TN}}}}{{\text{TN + FP}}} \times 100\%$$

Table 1 summarizes the method comparison and ablation experiment. We evaluated the classic classification of deep neural networks and several attention-oriented architectures. It can be observed that our proposed MCRFNet has noticeably higher performance than other models, achieving Acc, PP, Se, and Sp values of 94.39%, 82.28%, 76.27% and 96.44% on the three datasets, respectively. Compared with classic models, our model is specially designed for COVID-19 lung sonogram images, and attention-guided architecture has advantages in ultrasound images. Attention-oriented models show better performance than classic models; however, no previous models have fused multimodal information or considered attention combining, which impacted their classification performance.

**Table 1:** Method comparison and ablation experiment on different datasets

Method	S (%)				S&M (%)				S&M&P (%)			
	Acc	PP	Se	Sp	Acc	PP	Se	Sp	Acc	PP	Se	Sp
Method comparison												
VGG [20]	93.15	73.07	74.62	96.09	89.87	76.36	73.05	94.06	88.81	73.5	67.37	93.19
ResNet [21]	93.19	73.56	75.55	96.06	90.63	77.77	74.94	94.51	89.41	74.56	68.78	93.56
ResNeXt (baseline) [22]	93.15	77.82	74.62	96.31	91.7	79.98	77.57	94.94	90.46	75.92	70.9	94.04
SENet-50 [23]	92.5	72.16	73.33	95.65	93.64	83.16	**83.74**	96.28	92.37	79.71	75.93	95.33
SKNet-50 [24]	92.8	**86.32**	76.97	95.79	92.12	80.45	78.63	95.4	91.03	77.21	72.55	94.55
Ablation experiment												
w/o. CRFA and w/o. fusion module	93.15	77.82	74.62	96.31	91.7	79.98	77.57	94.94	90.46	75.92	70.9	94.04
w/o. CRFA module	93.19	78.09	75.55	96.34	92.12	81.21	78.63	95.17	90.93	77.14	71.94	94.3
MCRFNet	**97.73**	85.72	**88.06**	**98.69**	**96.25**	**87.2**	83.59	**97.74**	**94.39**	**82.28**	**76.27**	**96.44**

*S* Stork dataset accuracy, *M* Mindray dataset accuracy, *P* Philips dataset accuracy, *Acc* accuracy, *PP* precision, *Se* sensitivity, *Sp* specificity

Table 1 shows the results of a comparison between models trained with or without the CRFA module and fusion module. Specifically, the model trained with the CRFA and fusion module yields a 0.028 improvement in Acc, a 0.0636 improvement in PP, a 0.0537 improvement in Se, and a 0.024 improvement in Sp, which greatly surpasses the baseline model.

**Fig. 1:**
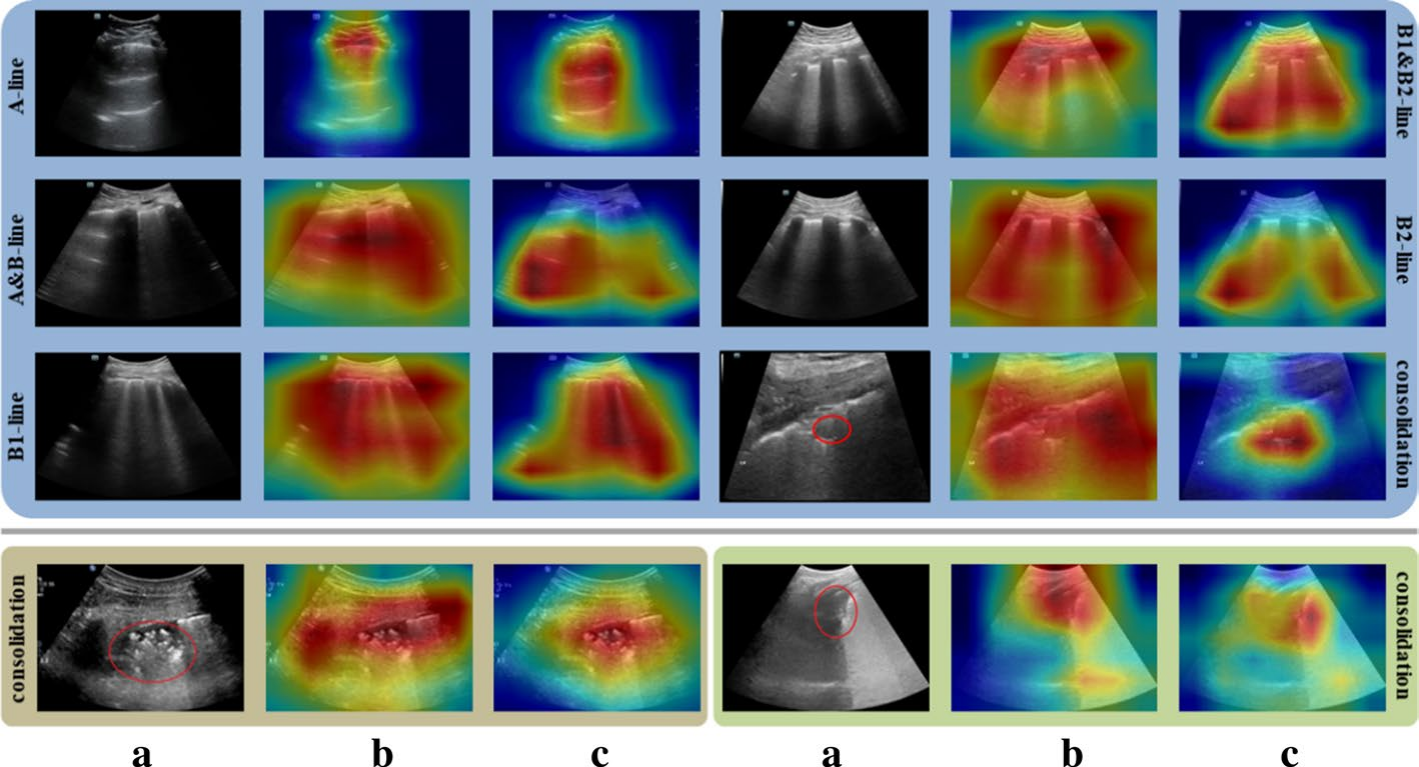
The Grad-CAM++ visualization results. The blue, red, and green backgrounds represent the Mindray dataset, Stork dataset, and Philips dataset, respectively. **a** Original input (the actual data are not marked with a red circle); **b** ResNeXt, and; **c** MCRFNet. The red circle is the lung consolidation area

To intuitively understand the fusion module, channel, and receptive field attention capability of MCRFNet, we used the Grad-CAM++ method to visualize the class activation mapping of the backbone and our proposed network [25]. Grad-CAM++ is commonly used to locate discriminative feature regions for perception, which makes the model interpretable. As shown in Fig. 1, the areas with bright colors indicate that the current region contributes the most to the classification. The results show that the backbone network focuses on the recognition of the B-line in a wider area, and our proposed network can better allow the network to focus on the line-shaped area or lung consolidation area. The samples are well recognized by ResNeXt, but there is still room for improvement. However, our MCRFNet can adaptively select the appropriate modal channel and suitable convolution kernel size. Finally, our MCRFNet performs better than ResNeXt due to the influence of the MCRF module.

**Fig. 2:**
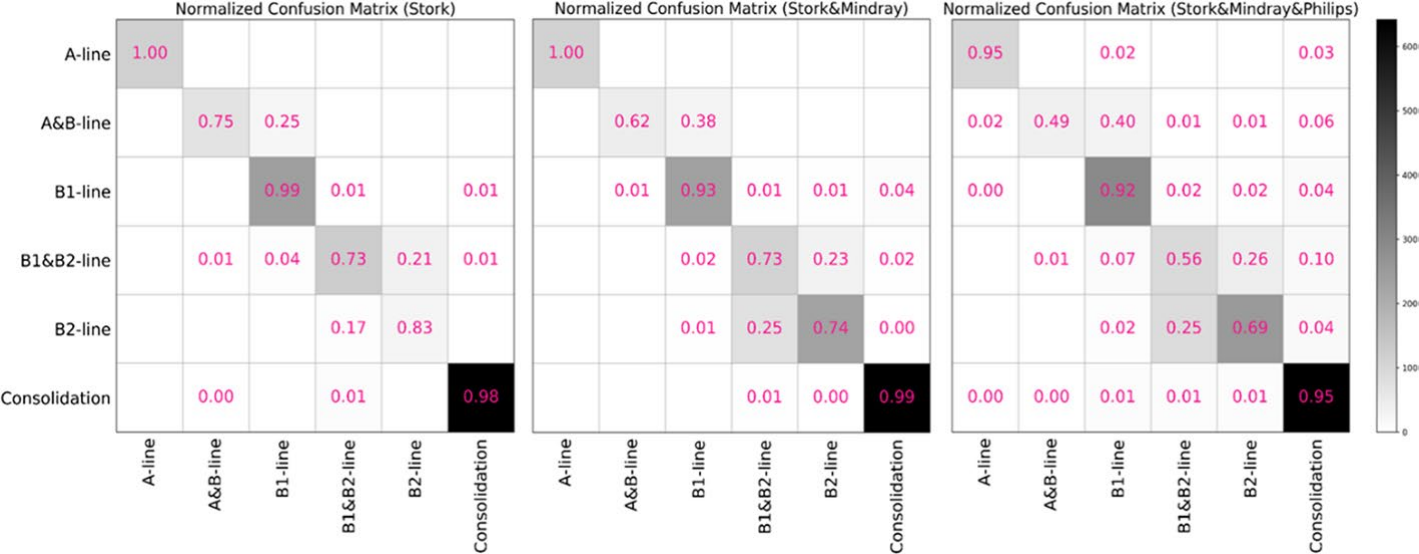
The normalized confusion matrixes of classification on three datasets by MCRFNet. The abscissa is the predicted label, and the ordinate is the true label

The normalized confusion matrixes of classification on three datasets by our proposed method are provided in Fig. 2. We observe that B2-line, B1&B2-line, and B1-line are partly misclassified as lower or higher level severity. Especially in the three datasets, misclassification is more obvious. The reasons for the performance degradation of multiple datasets will be discussed in the “Discussion” section. In short, the classification accuracy of the A-line and consolidation of the three datasets can reach nearly 100%. Misclassification generally occurs in the fusion classification of two categories: A&B-line and B1&B2-line.

### Distribution of datasets

**Fig. 3:**
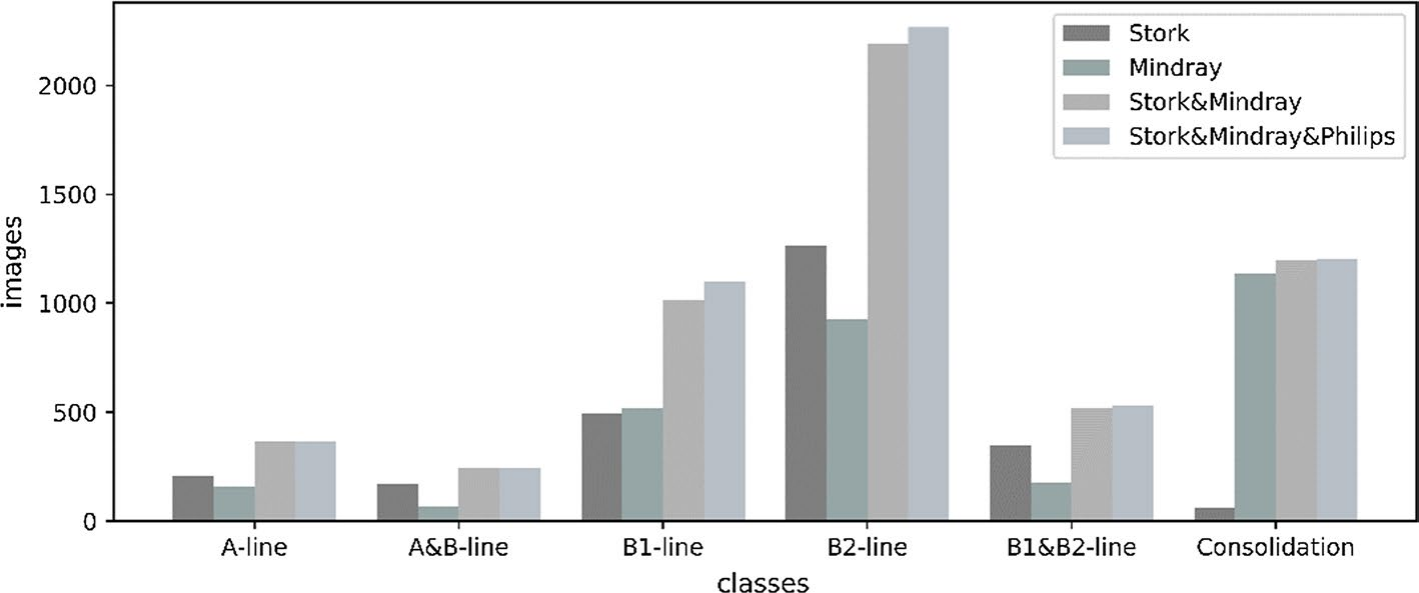
Distribution of expert manual labels of 5704 ultrasound images in three datasets

Figure 3 depicts the distribution of expert manual label classification of 5704 ultrasound images of 108 cases in three datasets. Specifically, the Stork, Mindray and Philips datasets have 2541 images of 43 cases, 2985 images of 51 cases, and 178 images of 14 cases, respectively, and we merged the first two and the first three datasets as two new datasets (Stork & Mindray, Stork & Mindray & Philips) to avoid imbalanced samples. These three datasets (Stork, Stork & Mindray, Stork & Mindray & Philips) are divided into training and test sets at a ratio of 2:1 for each category. The number of cases in the training set of the three datasets are: 29, 63, and 72, and the number of cases in the test set are 14, 31, and 36. We solved the overfitting problem by randomly flipping images, early stopping strategy, dropout, and L2 regularization.

### Evaluation of the trend in the degree of lung involvement

**Fig. 4:**
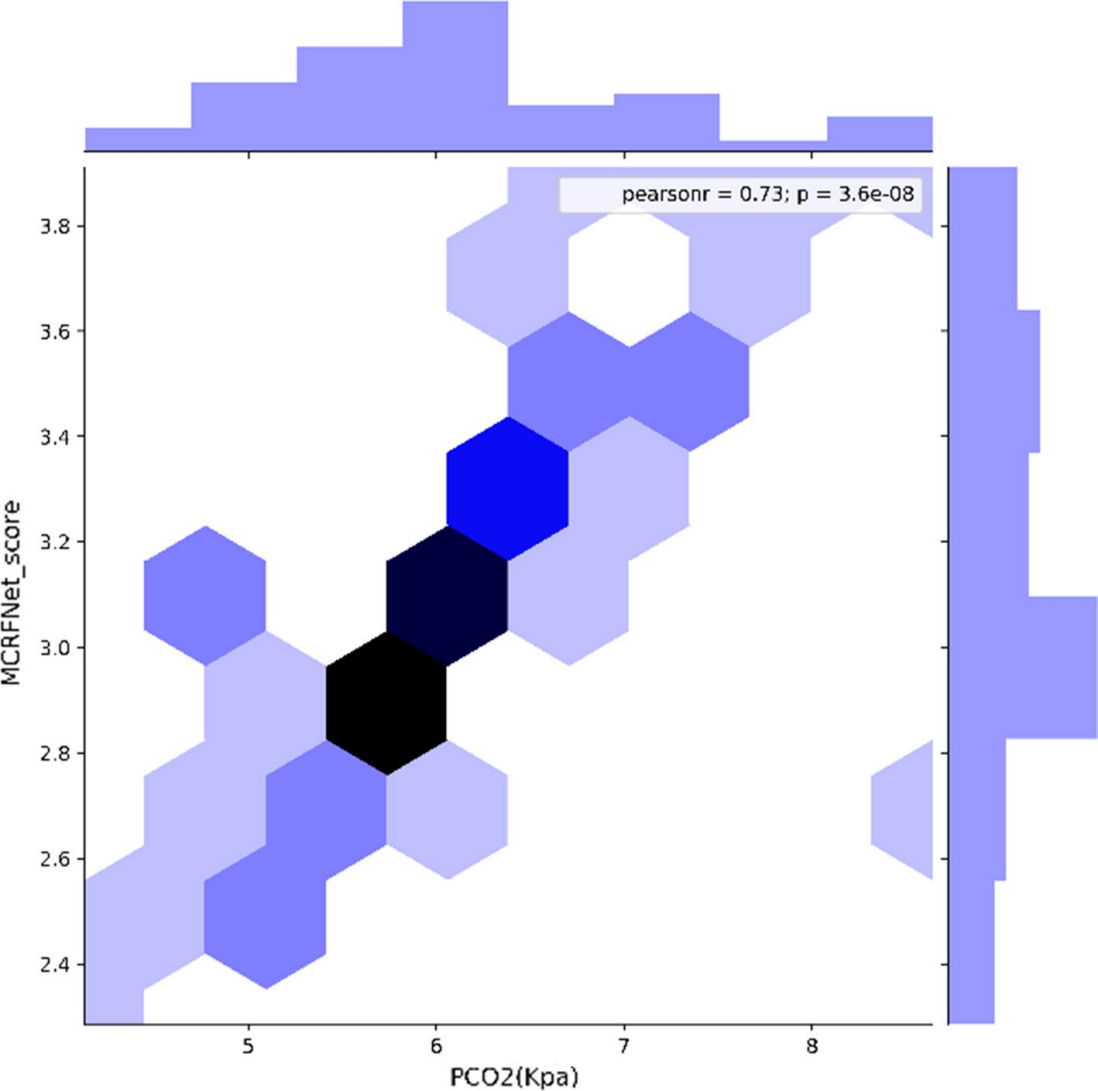
Scatter plot between the classification score of MCRFNet and CO2 partial pressure (PCO2)

After obtaining the trained MCRFNet, we independently tested the videos of 8 patients (additional collected data), which were examined multiple times (4 times or more) from the initial diagnosis to rehabilitation, and we performed classification according to the method in the *Establishment of Scoring Standards* section. CO2 partial pressure (PCO2) is a great indicator of respiratory function and is closely related to acid–base homeostasis, reflecting the amount of acid in the blood. The correlation between the score obtained and PCO2 was analyzed by Pearson correlation analysis, and the correlation is shown in Fig. 4. The Pearson correlation coefficient was 0.73 (P < 0.001). In the graph, darker colors indicate a higher frequency of occurrence. The graph shows that the score of MCRFNet is in the range of 2.7–3.4, which has a higher correlation with PCO2.

**Fig. 5:**
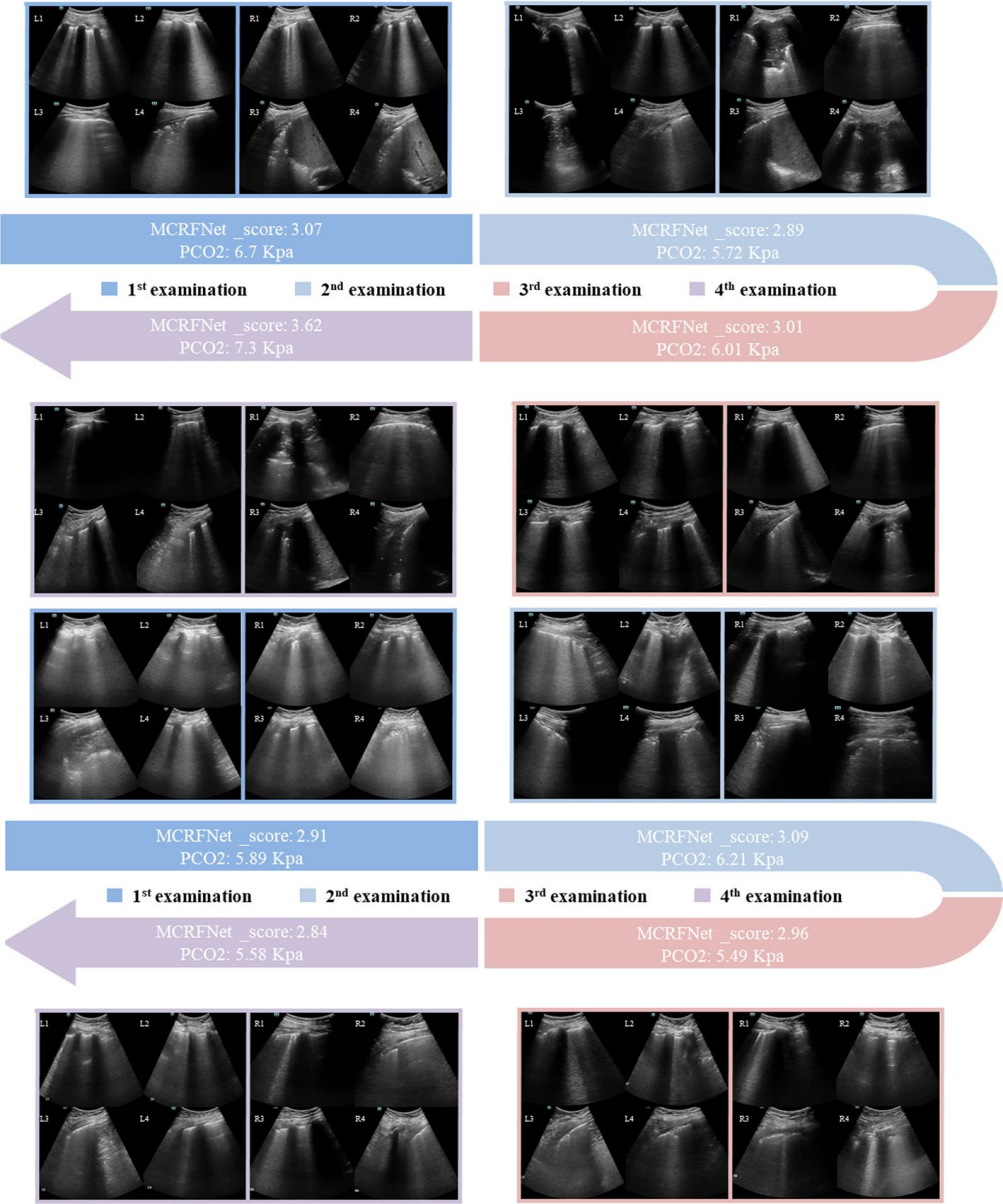
Two patients had multiple examinations of the MCRFNet score and PCO2 from the initial diagnosis to rehabilitation

In addition, two patients with multiple examinations of the MCRFNet score and PCO2 are shown in Fig. 5. We followed the three lines of the parasternal line (PSL), anterior axillary line (AAL), and posterior axillary line (PAL) in the reference [26] to divide the left and right sides of the lungs into four areas (L1–L4 and R1–R4). Only one picture is shown in the figure, but in the actual scoring, we averaged the scores of multiple pictures after framing to obtain the specific score in the figure.

In short, our classification and scoring system not only reflects the degree of lung involvement of a patient, but also helps doctors combine this score with other indicators to evaluate the patient’s lung disease and even the entire person’s condition. It is more beneficial to use this scoring system to improve the nursing level of patients with COVID-19 pneumonia and enhance their support for the clinical decision-making process for the management cascade.

## Discussion

The current challenge of COVID-19 is not its diagnosis, but its impact on internal organs. Recently, an increasing number of COVID-19-related diagnostic and segmentation studies have been published, but the damaging effects of COVID-19 on the internal organs of the human body are more important. In this paper, we proposed a novel multimodal channel and receptive field attention network (MCRFNet) for assessing lung damage in COVID-19 patients.

Rouby et al. assessed lung involvement by scoring eight areas’ sonograms [27], while the sonograms need to be manually identified by doctors, which is time-consuming and labor-intensive. There are also some studies on the automatic classification of sonograms, but most of them are only for detecting the B-line of sonograms [28, 29]. Our MCRFNet can achieve fully automatic assessment of lung involvement in COVID-19 patients. The lung ultrasound images of these patients were classified into six types of sonograms, and the classification results were quantitatively scored to obtain the total scores of 8 regions. Then, a correlation analysis between the scores and PCO2, which is the most relevant to lung involvement, was obtained. Finally, a Pearson correlation coefficient of 0.73 was calculated, indicating that our classification scores can reflect the lung involvement of COVID-19 patients. It is useful to choose the correct treatment method based on the severity of the situation.

**Fig. 6:**
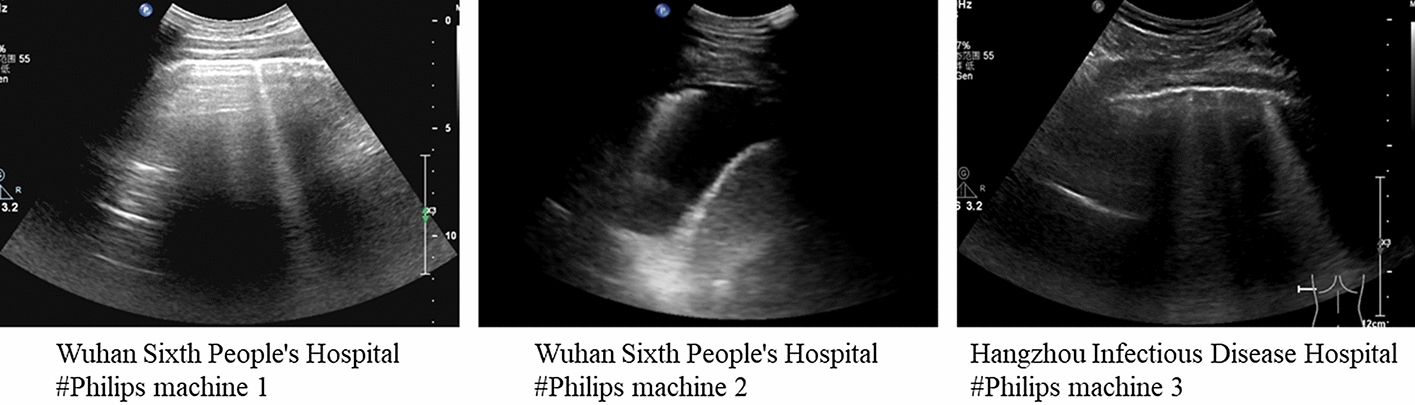
Comparison of B1-line of Philips dataset from three different machines

The reason for the performance decline after adding the Philips dataset is shown in Fig. 6. The Philips dataset comes from three different machines in two centers. Due to its contrast and resolution disparity with the Stork and Mindray datasets, our model misclassified some images into incorrect categories. It also means that the robustness and cross-domain adaptability of our model are not perfect; this limitation serves as a direction for future improvement. In terms of this problem, to make the classification model more robust, we used traditional methods to extract shallow features that are not sensitive to imaging parameters and observed great performance.

Some attempts were made to verify the performance of the model. We tried to train with data from a single center, while data from another center were used for independent testing. However, the Stork dataset has only 58 consolidation images; if we use the trained Stork model to predict Mindray's consolidation data (1136 images), the accuracy of independent testing will be greatly reduced in this category of classification and vice versa.

For further study, we consider applying the lung consolidation attention area of our model to segment the lung consolidation part. The data in this experiment are obtained by an oblique scan, our model may be used for longitudinal scan in further study, making the model robust to the scanning method. We will try to use the patient's ultrasound video as input, and complete the direct assessment of the patient's lung involvement through three-dimensional convolutional neural network (3D-CNN) [30] or long short-term memory (LSTM) [31].

## Conclusions

In this paper, we proposed a novel classification network named MCRFNet that utilizes multimodal fusion and channel and receptive field attention to classify lung sonograms. In addition, we scored the predicted categories that reflect the degree of lung involvement in the patient and helped doctors to combine other indicators to assess disease trends in COVID-19 patients.

## Methods

### Ultrasound data acquisitions

**Fig. 7:**
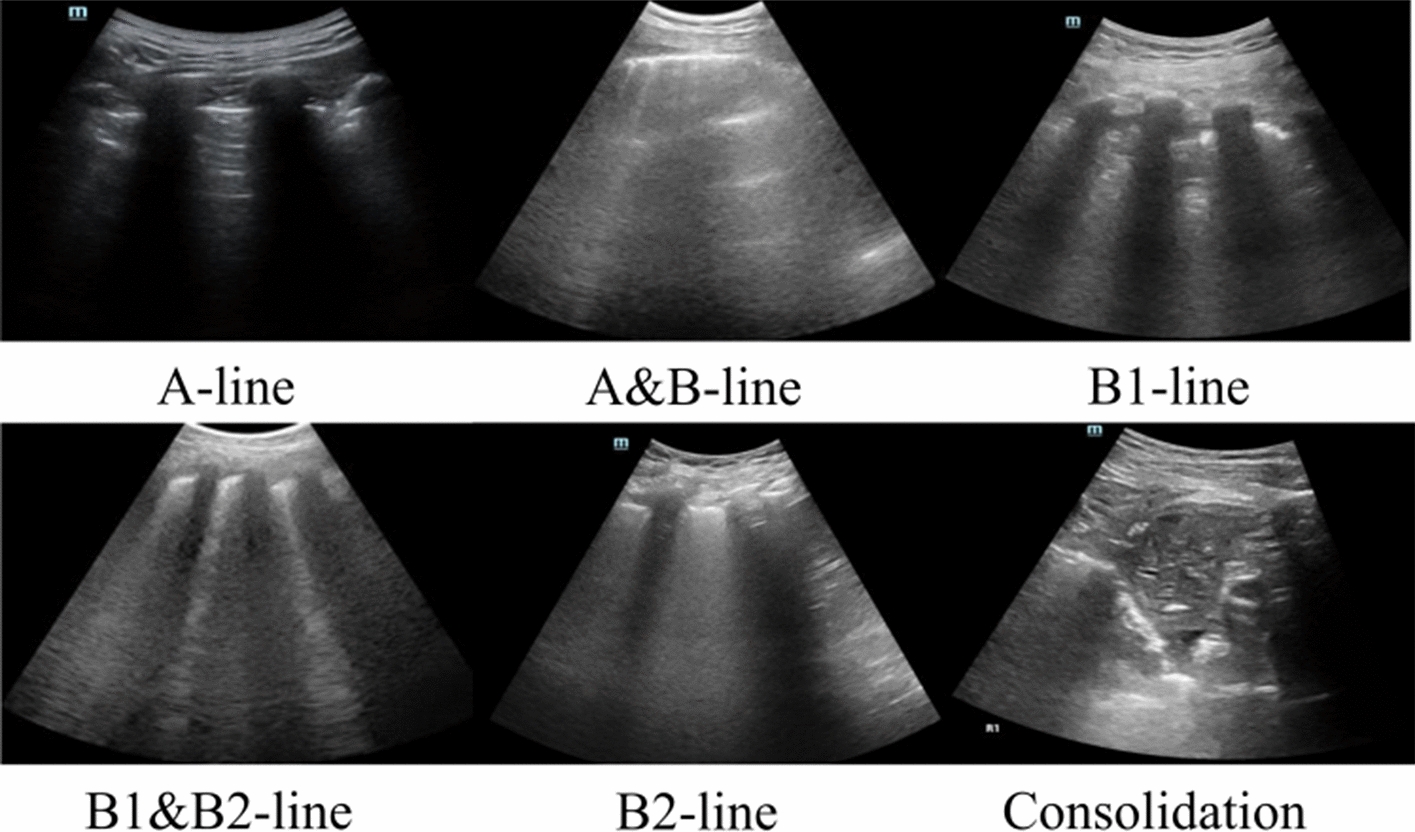
Different ultrasound sonograms in lung examination

In ultrasound imaging, the degree of lung involvement is related to several typical sonograms. The A-line is a horizontal reverberation artifact of the pleura caused by multiple reflections, representing the normal lung surface [32]. The B-line represents the interlobular septum, which is denoted by a discrete laser-like vertical hyperechoic artifact that spreads to the end of the screen, and it can be represented as the B1-line [33]. The fusion B-line is a sign of pulmonary interstitial syndrome, which shows a large area filled with the B-line in the intercostal space, and it can be represented as the B2-line [26]. Pulmonary consolidation is characterized by a liver-like echo structure of the lung parenchyma, with a thickness of at least 15 mm [27], as shown in Fig. 7.

We used three datasets from four medical centers to build and evaluate the model: ultrasound images collected by the Stork ultrasound system (Stork Healthcare Co., Ltd. Chengdu, China) at Ruijin Hospital, Mindray ultrasound system (Mindray Medical International Limited, Shenzhen, China) at Shanghai Public Health Center, Philips ultrasound system (Philips Medical Systems, Best, the Netherlands) at Wuhan Sixth People's Hospital and Hangzhou Infectious Disease Hospital. The Stork dataset was collected with an H35C (2–5 MHz) convex array transducer, the Mindray dataset with an SC5-1 (1–5 MHz) convex array transducer, and the Philips dataset with an Epiq 5, Epiq 7 C5-1 (1–5 MHz) convex array transducer.

### Multimodal generation and fusion

**Fig. 8:**
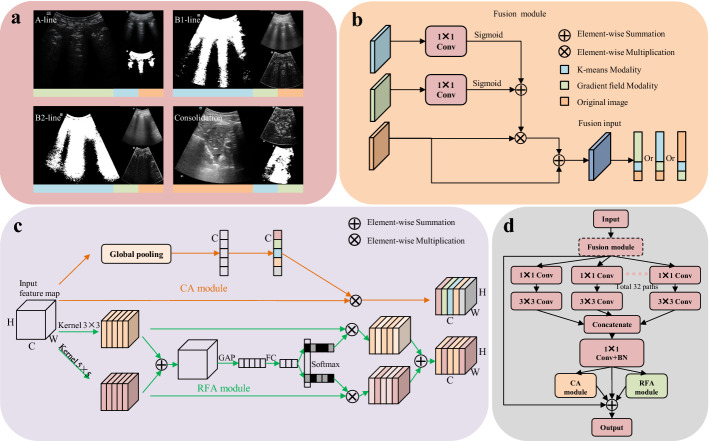
**a** Generated gradient field and K-means clustering modalities. The largest picture represents the most sensitive modal. **b** The proposed fusion module. **c** Details of CA and RFA module. **d** The MCRF block is integrated with a ResBlock in ResNeXt. The fusion module is only used in the first ResNeXt layer

According to doctors’ experience in recognizing sonograms, parallel echo rays of the A-line, beam-like echo rays of the B-line, and the accumulation of exudate of lung consolidation are used as markers for classification. The gradient field is highly sensitive to the parallel echo rays of the A-line, and K-means clustering can better highlight the beam-like echo rays of the B-line [28]. As shown in Fig. 8a, we produced the gradient field and K-means clustering images as two new modalities for extracting shallow features.

There are many methods to fuse multimodal inputs, and concatenate-based fusion is an intuitive fusion method [34], but this method is more suitable for situations where each modality is equally important for classification. Extracting features first and then concatenating is also a very popular fusion method [35], while the number of parameters and GPU memory limit its application. In this paper, we proposed a brand-new fusion network, as shown in Fig. 8b; this network used the minimum network parameters to achieve multimodal automatic weight distribution, thus underlining the embedding of the other two modalities on the original image. Two 1 × 1 convolutions were used to update the weights of the K-means modality and gradient field modality in the easiest way. After elementwise summation, we highlighted it on the original image by elementwise multiplication with the original image and finally added it to the original image to obtain the final fusion input.

### ResNeXt with CRF attention block for classification

Shallow features were extracted by the traditional methods in “Multimodal generation and fusion” section. To extract deep features more effectively, we chose deep and wide ResNeXt as the backbone network for classification. ResNeXt [22] is a combination of ResNet [21] and Inception [36], which improves accuracy through wider or deeper networks. Each of its blocks is a measurable dimension in addition to the width and depth dimensions. It inherits the strategy of repeating layers of ResNet, but increases the number of paths and uses split conversion and merge strategies in a simple and scalable manner. ResNeXt with the CRF attention building block is shown in Fig. 8d. Our whole network replaces the building block in ResNeXt with our CRF attention building block. In detail, there is one first layer and three residual layers in our network, and every first layer and residual layer has one and three grassroots CRFA building blocks, respectively.

The CRF attention module comprised channelwise and receptive field attention modules, denoted as CA and RFA, respectively (Fig. 8c). The CA module attempts to assist the learning of layer-specific features and explores channelwise dependencies for the selection of useful features. Specifically, given an intermediate input feature channel set $U \in {R^{H \times W \times C}}$, a squeeze operation is performed on the input image $F_{sq} (U)$, that is, global average pooling (GAP), to encode the entire spatial feature on a channel as a global feature:

5 $$F_{sq} (U) = \frac{1}{W \times H}\sum\limits_{i = 1}^{W} {\sum\limits_{j = 1}^{H} {U(i,j), \, U \in R^{W \times H \times C} } }$$

The squeeze operation obtains the global description feature, and another operation is required to capture the relationship between the channels, namely, the excitation operation $F_{ex} (F_{sq} (U))$:

6 $$\begin{gathered} F_{ex} (F_{sq} (U)) = \sigma \left( {W_{2} \delta \left( {W_{1} F_{sq} \left( U \right)} \right)} \right), \hfill \\ \delta \left( x \right) = \max \left( {0,x} \right),\sigma (x) = \frac{1}{{1 + e^{ - x} }} \hfill \\ \end{gathered}$$

where $\delta ( \cdot )$ and $\sigma ( \cdot )$ are the ReLU activation and sigmoid function, respectively. ReLU is a ramp function which has gradient one for positive inputs and zero for negative inputs. Sigmoid function maps the input from 0 to 1. $W_{1} \in R^{{\frac{c}{16} \times c}}$ and $W_{2} \in R^{{\frac{c}{16} \times c}}$ are the learning weights of the two fully connected layers. The excitation operation can learn the nonlinear relationship between channels. Finally, the learned activation value of each channel (sigmoid activation) is multiplied by the original feature on $U$:

7 $$\hat{U}_{C} = F(U,F_{ex} (F_{sq} (U))) = U \cdot F_{ex} (F_{sq} (U)),U \in R^{W \times H \times C}$$

given the same input feature channel set $U \in R^{H \times W \times C}$, we first conducted two transformations $\tilde{F}:U \to \tilde{U} \in R^{H \times W \times C}$ and $\hat{F}:U \to \hat{U} \in R^{H \times W \times C}$ with kernel sizes of 3 and 5, respectively. Then, the results of multiple branches are combined by summing the elements as follows:

8 $$U{ = }\tilde{U}{ + }\hat{U}$$

For the output features $\tilde{U}$ and $\hat{U}$, squeeze and excitation are performed, respectively, as in Eq. 2. Additionally, we used soft attention across channels to select different spatial scales of information, which is guided by the compact feature descriptor **z**:

9 $$\hat{U}_{RF} = \frac{{e^{{A_{c} z}} }}{{e^{{A_{c} z}} + e^{{B_{c} z}} }}F_{ex} (F_{sq} (\tilde{U})) + (1 - \frac{{e^{{A_{c} z}} }}{{e^{{A_{c} z}} + e^{{B_{c} z}} }})F_{ex} (F_{sq} (\hat{U})),U \in R^{W \times H \times C}$$

where $A,B \in R^{{\frac{C}{16} \times C}}$. The final feature map of RFA is obtained through the attention weights on various kernels as in the above equation.

With the CA and RFA modules, the CA and RFA results are further integrated with the add operation, as shown in Fig. 6d:

10 $$\overline{U} = \hat{U}_{C} + \hat{U}_{RF}$$

Detailed procedures are as follows: (1) extract the most common 6 types of datasets in Fig. 5 from the training set in equal proportions randomly to avoid an imbalanced sample and ensure that each category can be learned. (2) Augment the data by rotation and normalize the intensity of the image. (3) Select the classifier with the best performance and test it on the test set to obtain the corresponding prediction results.

### Establishment of scoring standards

We predicted the patient's per part ultrasound video of multiple examinations through the trained MCRFNet and classified and scored sonograms according to the paper [37]. A-line indicates that the patient is normally ventilated, with a score of 0; A&B-line indicates that the patient has mild lung ventilation loss, with a score of 1; B1-line indicates that the patient has moderate lung ventilation loss, with a score of 2; B1&B2-line indicates that the patient has severe lung loss of ventilation, with a score of 2.5; B2-line indicates that the patient has very severe loss of lung ventilation, with a score of 3; consolidation indicates that the patient has a solid lung change characterized by dynamic air bronchial signs, with a score of 4. After the classification result is quantified, the sum is divided by all the frames to obtain the final lung function severity score, which is 0 to 4.

### Training strategy

For the Stork, Mindray, Stork & Mindray, and Stork & Mindray & Philips datasets, we used an independence test to verify the performance of the classifier. All the images were resized to 128 × 128, and a training batch consisted of 8 randomly selected images. We regularized the model by using dropout during training, and the neural network parameters were then trained by maximizing log-likelihood using the momentum optimizer with an initial learning rate of 0.1. Then, every 30 epochs, the learning rate dropped by 10 times, stochastically minimizing the cross-entropy between annotated labels and predictions. Our experiments were implemented using TensorFlow on a PC with an Intel Xeon E5, 64G RAM, Nvidia TITAN Xp 12G.

## Data Availability

The datasets used and analyzed during the current study are available from the corresponding author on reasonable request.

## Abbreviations

LUS: Lung ultrasound
COVID-19: Novel coronavirus 2019
RT-PCR: Reverse transcription-polymerase chain reaction
CT: Computed tomography
VAP: Ventilator-associated pneumonia
PCO2: CO2 partial pressure
PSL: Parasternal line
POI: Points of interest
AAL: Anterior axillary line
PAL: Posterior axillary line
